# First isolation and whole genome characterization of porcine deltacoronavirus from pigs in Peru

**DOI:** 10.1111/tbed.14489

**Published:** 2022-03-15

**Authors:** Juan A. More‐Bayona, Mercy Ramirez‐Velasquez, Ben Hause, Eric Nelson, Hermelinda Rivera‐Geronimo

**Affiliations:** ^1^ Laboratory of Virology Faculty of Veterinary Medicine Universidad Nacional Mayor de San Marcos Lima Peru; ^2^ Department of Veterinary and Biomedical Sciences South Dakota State University Brookings South Dakota USA; ^3^ Cambridge Technologies Worthington Minnesota USA

**Keywords:** diarrhea, emerging diseases, PDCoV isolation, Peru, porcine deltacoronavirus, veterinary epidemiology, whole genome sequencing

## Abstract

Porcine deltacoronavirus is a newly emergent enteric pathogen affecting swine farms worldwide. It has been detected in several countries in Europe, Asia and North America; yet, it has not been reported in South America. In November 2019, an enteric disease outbreak in a pig farm located in San Martin, Peru, was reported along with submission of three intestinal samples from pigs who succumbed to the disease. Samples were processed for molecular detection by qRT‐PCR, viral isolation and further sequencing analysis. A taqman‐based RT‐PCR was performed to differentiate among the most relevant swine enteric coronaviruses described to date. All samples were positive to porcine deltacoronavirus with a cycle threshold (Ct) value between 9 and 14, revealing a high viral load, while testing negative to porcine epidemic diarrhea and transmissible gastroenteritis viruses. Following detection, viral isolation was performed using PK‐15 and Vero cell lines. After 5 days of inoculation, no cytopathic effect was observed. A second blind passage allowed the observation of cytopathic effect on PK‐15 cells, while it remained absent in Vero cells. A fluorescence test using an anti‐N monoclonal antibody confirmed viral replication. One sample was processed for whole genome sequencing (WGS). In short, raw reads were imported into CLC genomics and assembled de novo. Out of 479k reads generated from the sample, 436k assembled into a 25,501 bp contig which was 99.5% identical to a reference porcine deltacoronavirus strain from the USA within the North American phylogroup. Yet, there are relevant differences at the nucleotide and amino acid levels compared with previously described porcine deltacoronavirus strains. Altogether, our findings represent the first report of porcine deltacoronavirus in South America, which provides information of its evolutionary origin. Thus, this study offers new insights into the molecular epidemiology of porcine deltacoronavirus infections in the swine industry.

## 1 INTRODUCTION

Coronaviruses comprise a large group of single‐stranded, positive‐sense RNA viruses that infect a broad range of species such as avian and mammals, including humans. Coronaviruses belong to the order *Nidovirales*, family *Coronaviridae*, subfamily *Coronavirinae*. These enveloped viruses are the largest RNA viruses identified to date ranging from 24 to 32 kb. Members of the subfamily *Coronavirinae* have been recently grouped into four genus as *Alphacoronaviruses*, *Betacoronaviruses*, *Gammacoronaviruses* and *Deltacoronaviruses* by the International Committee for Taxonomy of Viruses (Lefkowitz et al., 2018; Woo et al., 2010). Interestingly, it appears that the first two groups have originated from bats, whereas the latter two emerged from wild birds (Woo et al., 2012).

Porcine deltacoronavirus (PDCoV) is an emergent virus that causes gastrointestinal disease such as diarrhea, vomiting, dehydration and death in young piglets representing a major threat to swine industry (Jung et al., 2015; Li et al., 2019; Zhang, 2016; Zhao et al., 2019). Although PDCoV by itself causes enteric disease, co‐infections with other coronaviruses such as porcine epidemic diarrhea virus (PEDV) and transmissible gastroenteritis virus (TGEV) or other viruses are commonly found (Ajayi et al., 2018; Mai et al., 2017; Marthaler et al., 2014a; Niederwerder, 2018; Song et al., 2015). In this context, PDCoV shows indistinguishable clinical signs from other forms of enteric disease such as PED, TGE and swine acute diarrhea syndrome. Thus, proper differential diagnostic relies on genetic detection‐based assays that offer a highly sensitive and specific method.

PDCoV has a unique genomic organization. Starting from 5′‐end, PDCoV has a 5′ untranslated region (UTR), replicase (ORF 1a/b), spike (S), envelope (E), membrane (M), non‐structural 6 (NS6), nucleocapsid (N), NS7 genes and 3′‐UTR (Woo et al., 2010; Zhang, 2016). Interestingly, PDCoV lacks ORF3 and NS1, present in other well‐known coronaviruses (Si et al., 2020). From these genes, S gene encodes a highly glycosylated protein responsible for binding, cell attachment and entry into target cells, and therefore highly immunogenic. Thus, S gene is commonly used for phylogenetic analysis and vaccine development.

The first identification of PDCoV dates back to 2012 in Hong Kong by Woo et al, followed by multiple outbreaks in the USA (Homwong et al., 2016; Marthaler et al., 2014b; Wang et al., 2014). Later, PDCoV was reported in Canada (Niederwerder, 2018), Korea (Jang et al., 2018; Lee & Lee, 2014; Lee et al., 2016), Japan (Suzuki et al., 2018), Thailand (Lorsirigool et al., 2017; Saeng‐Chuto et al., 2016) and Vietnam (Le et al., 2018; Saeng‐chuto et al., 2019). More recently, PDCoV was detected in Mexico (Pérez‐Rivera et al., 2019). In South America, coronavirus infections in porcine are commonly reported. For instance, multiple studies revealed that PEDV and TGEV are present in several countries such as Colombia (Piñeros & Mogollón Galvis, 2015), Ecuador (Barrera et al., 2017) and Peru (Castro‐Sanguinetti et al., 2017). However, PDCoV has never been reported in South America. Hence, we report the first isolation and whole genome sequencing of PDCoV in Peru, providing new insights into the molecular epidemiology of this emerging disease in swine.

## 2 MATERIALS AND METHODS

### 2.1 Samples and RNA extraction

**FIGURE 1:**
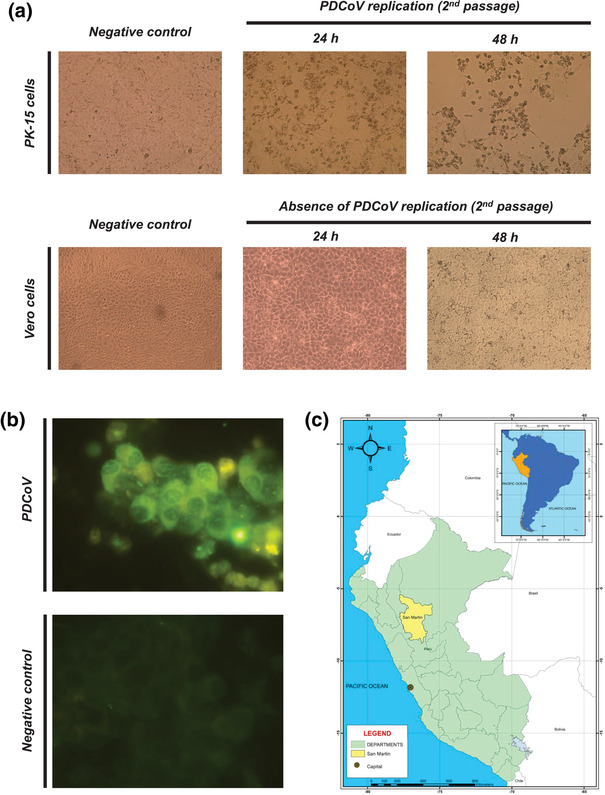
PDCoV isolation in PK‐15 cells and geographical map indicating locations where the outbreak was reported. Following PDCoV detection, intestinal content was filtered, and TPCK‐trypsin treated for isolation in cell lines known to be permissible for PDCoV replication. Following 5 days of inoculation, no cytopathic effect (cpe) was evidenced under light microscopy in both cell lines. A second passage evidenced cpe after 24 h, with initial cell rounding and monolayer disruption in PK‐15 cells but absent in Vero cells. Following 48 h of second passage, cpe was evidenced in almost 70% of cell monolayer, characterized by cell rounding, cell detaching, pyknosis in PK‐15 cell lines but no cytopathic effect was detected in Vero cell lines (a). Moreover, an immunofluorescence test with a PDCoV nucleoprotein monoclonal antibody confirmed viral replication in PK‐15 infected cells (b). A representative map of South America (blue) highlighting Peru (orange) is shown in the upper right side. Additionally, a geographical map of San Martin department (yellow) located in the north of Peru (light green) is represented below (c)

In November 2019, a report of an enteric clinical disease outbreak in a farm located in San Martin, Peru (see Figure 1(c)) was made by a local veterinary professional to the National animal health authorities (SENASA). The report indicated high morbidity in young piglets. Intestinal samples were submitted by the farm professionals to the Laboratory of Virology in the Faculty of Veterinary Medicine at the Universidad Nacional Mayor de San Marcos in Lima, Peru, for testing and detection of PEDV, TGEV and PDCoV by qRT‐PCR. Intestinal content (1 ml) was processed accordingly. In short, samples were diluted in 5 ml of phosphate buffer solution and centrifuged at 250 g for 5 min. Following centrifugation, 1 ml of supernatant was processed for RNA extraction using the QIAmp viral RNA mini kit (Qiagen), following manufacturer's specifications.

### 2.2 PCR differentiation and detection

Samples were assayed by qRT‐PCR to differentiate three main enteric coronaviruses affecting pigs as PEDV, TGEV and PDCoV. Thus, we used the EZ‐PED/TGE/PDCoV MPX 1.1 kit (Tetracore) following manufacturer's specifications.

### 2.3 Viral isolation

For viral isolation, we tested two different cell lines known to support coronavirus replication. We used PK‐15 and Vero cells that were plated in 24‐well plates at 90% of confluence. PK‐15 cells were kindly provided by Dr. Jhon Pasick from the Canadian Food Inspection Agency (Ontario, Canada) and Vero cells by the Naval Medical Research Unit Six (NAMRU‐6). Briefly, 100 μl of intestinal content was filtered and treated with TPCK‐trypsin (10 μg/ml) to allow ligand cleavage for further virus attachment to cell receptors. Treated samples were inoculated and incubated for 1 h, followed by replacement of maintenance media with trypsin. Cells were incubated for 5 days at 37°C with 5% CO_2_, with a second blind passage. Cytopathic effect was evaluated daily, and pictures recorded under light microscopy (Leica Microsystems) using a Leica MC170 HD camera (Leica Microsystems). Following isolation, we performed an immunofluorescence test using a PDCoV anti‐nucleoprotein monoclonal antibody (SD55‐197), kindly provided by Drs. Eric Nelson and Steven Lawson from the South Dakota State University (South Dakota, USA), to confirm the presence of the PDCoV.

### 2.4 Whole genome sequencing

Viral RNA was purified from one sample and processed for next‐generation sequencing (Illumina, Inc), following manufacturer's specifications. Reads were imported into CLC genomics and assembled de novo. Whole genome (*n* = 44) and ORF 1a/b (*n* = 23) sequences of PDCoV were obtained from GenBank for phylogenetic analysis. For analysis based on S gene, we also included those from Mexico (*n* = 61). General information of nucleotide sequences used is listed in Table 1.

**TABLE 1:** Nucleotide sequences for phylogenetic analysis

	Full name	Short name	Country	Length (nt)	Accession number
1	Porcine deltacoronavirus strain USA/NorthCarolina452/2014, complete genome	PDCoV/USA/NorthCarolina452/2014	USA	25394	KR265858.1
2	Porcine deltacoronavirus strain USA/Minnesota/2013, complete genome	PDCoV/USA/Minnesota/2013	USA	25394	KR265853.1
3	Porcine deltacoronavirus strain USA/Illinois449/2014, complete genome	PDCoV/USA/Illinois449/2014	USA	25394	KR265852.1
4	Porcine deltacoronavirus strain USA/Minnesota159/2014, complete genome	PDCoV/USA/Minnesota159/2014	USA	25401	KR265859.1
5	Deltacoronavirus PDCoV/USA/Illinois134/2014 from USA, complete genome	PDCoV/USA/Illinois134/2014	USA	25404	KJ601778.1
6	Porcine deltacoronavirus strain USA/Nebraska210/2014, complete genome	PDCoV/USA/Nebraska210/2019	USA	25404	KR265861.1
7	Porcine deltacoronavirus genomic RNA, complete genome, strain: YMG/JPN/2014	PDCoV/JPN/YMG/2014	Japan	25362	LC260044.1
8	Porcine deltacoronavirus strain USA/Michigan448/2014, complete genome	PDCoV/USA/Michigan448/2014	USA	25394	KR265850.1
9	Porcine deltacoronavirus strain USA/Michigan447/2014, complete genome	PDCoV/USA/Michigan447/2014	USA	25393	KR265849.1
10	Deltacoronavirus PDCoV/USA/Ohio137/2014 from USA, complete genome	PDCoV/USA/Ohio137/2014	USA	25404	KJ601780.1
11	Porcine deltacoronavirus strain USA/Indiana453/2014, complete genome	PDCoV/USA/Indiana453/2014	USA	25394	KR265851.1
12	Porcine deltacoronavirus strain USA/Minnesota455/2014, complete genome	PDCoV/USA/Minnesota455/2014	USA	25394	KR265855.1
13	Porcine deltacoronavirus strain USA/Minnesota454/2014, complete genome	PDCoV/USA/Minnesota454/2014	USA	25394	KR265854.1
14	Porcine deltacoronavirus strain USA/Arkansas61/2015, complete genome	PDCoV/USA/Arkansas61/2015	USA	25398	KR150443.1
15	Deltacoronavirus PDCoV/USA/Illinois121/2014 from USA complete genome	PDCoV/USA/Illinois121/2014	USA	25406	KJ481931.1
16	Porcine deltacoronavirus 8734/USA‐IA/2014 complete genome	PDCoV/USA‐IA/8734	USA	25422	KJ567050.1
17	Porcine deltacoronavirus strain OH‐FD22 P7 complete genome	PDCoV/USA/OH‐FD22	USA	25438	MZ291567.1
18	Porcine deltacoronavirus strain SHJS/SL/2016, complete genome	PDCoV/SHJS/SL/2016	China	25414	MF041982.1
19	Porcine deltacoronavirus strain CH/SXD1/2015, complete genome	PDCoV/CH/SXD1/2015	China	25419	KT021234.1
20	Porcine deltacoronavirus strain CHN‐LYG‐2014, complete genome	PDCoV/CHN‐LYG/2014	China	25370	KU665558.1
21	Porcine deltacoronavirus isolate PDCoV/CHJXNI2/2015, complete genome	PDCoV/CHJXNI2/2015	China	25438	KR131621.1
22	Porcine deltacoronavirus isolate CHN‐AH‐2004, complete genome	PDCoV/CHN‐AH/2004	China	25420	KP757890.1
23	Porcine deltacoronavirus strain CH/Hunan/2014 complete genome	PDCoV/CH/Hunan/2014	China	25413	KY513724.1
24	Porcine deltacoronavirus PDCoV/CH/SXD1/2015	PDCoV/CH/SXD1/2015	China	25419	KT021234.1
25	Porcine deltacoronavirus isolate 104–553 complete genome	PDCoV/104‐553	China	25418	MW854634.1
26	Porcine deltacoronavirus strain CHN/GS/2017/1 complete genome	PDCoV/CHN/GS/2017	China	25420	MF642324.1
27	Porcine deltacoronavirus strain CHN/QH/2017/1 complete genome	PDCoV/CHN/QH/2017	China	25420	MF642325.1
28	Porcine deltacoronavirus isolate CHN‐HN‐1601 complete genome	PDCoV/CHN/HN1601/2017	China	25419	MG832584.1
29	Porcine deltacoronavirus isolate CHN/Sichuan/2019 complete genome	PDCoV/ CHN/Sichuan/2019	China	25380	MK993519.1
30	Porcine deltacoronavirus isolate CH/JXJGS01/2016 complete genome	PDCoV/CH/JXJGS01/2016	China	25446	MK625641.1
31	Porcine deltacoronavirus isolate P1 16 BTL 0115/PDCoV/2016/Lao complete genome	PDCoV/Lao/2016	Lao	25405	KX118627.1
32	Porcine deltacoronavirus strain PDCoV/Swine/Vietnam/HaNoi6/2015, complete genome	PDCoV/Vietnam/HaNoi6/2015	Vietnam	25406	KX834351.1
33	Porcine deltacoronavirus strain PDCoV/Swine/Vietnam/Binh21/2015, complete genome	PDCoV/Vietnam/Binh21/2015	Vietnam	25406	KX834352.1
34	Porcine deltacoronavirus strain PDCoV/Swine/Thailand/S5015L/2015, complete genome	PDCoV/Thailand/S5015L/2015	Thailand	25405	KU051649.1
35	Porcine deltacoronavirus strain PDCoV/Swine/Thailand/S5011/2015, complete genome	PDCoV/Thailand/S5011/2015	Thailand	25405	KU051641.1
36	Porcine deltacoronavirus isolate KNU16‐07 complete genome	PDCoV/SouthKorea/KNU16‐07	South Korea	25422	KY364365.1
37	Porcine deltacoronavirus isolate KNU16‐11 complete genome	PDCoV/SouthKorea/KNU16‐11	South Korea	25419	KY926512.1
38	Porcine deltacoronavirus genomic RNA complete genome strain: IWT/JPN/2014	PDCoV/JPN/IWT/2014	Japan	25362	LC260041.1
39	Porcine deltacoronavirus genomic RNA complete genome strain: MYZ/JPN/2014	PDCoV/JPN/MYZ/2014	Japan	25362	LC260042.1
40	Porcine deltacoronavirus genomic RNA complete genome strain: AKT/JPN/2014	PDCoV/JPN/AKT/2014	Japan	25362	LC260038.1
41	Porcine deltacoronavirus genomic RNA complete genome strain: OKN/JPN/2014	PDCoV/JPN/OKN/2014	Japan	25362	LC260043.1
42	Porcine deltacoronavirus isolate PDCoV/Haiti/Human/0256‐1/2015 complete genome	PDCoV/Haiti/Human/0256‐1/2015	Haiti	25447	MW685623.1
43	Porcine deltacoronavirus isolate PDCoV/Haiti/Human/0329‐4/2015 complete genome	PDCoV/Haiti/Human/0329‐4/2015	Haiti	25444	MW685624.1
44	Porcine deltacoronavirus isolate PDCoV/Haiti/Human/0081‐4/2014 complete genome	PDCoV/Haiti/Human/0081‐4/2014	Haiti	25444	MW685622.1
45^†^	Porcine deltacoronavirus isolate CD‐HNZK‐02 S protein gene complete cds	PDCoV/CD‐HZNK‐02	China	3480	MZ326690.1
46^†^	Porcine deltacoronavirus strain CC‐HNZK‐02 S protein gene complete cds	PDCoV/CC‐HNZK‐02	China	3480	MK248485.1
47^†^	Porcine deltacoronavirus isolate SD‐01‐2018 spike glycoprotein gene complete cds	PDCoV/SD/01/2018	China	3480	MN173803.1
48^†^	Porcine deltacoronavirus strain CH‐XS‐2018 spike protein gene complete cds	PDCoV/CH/XS/2018	China	3480	MK040452.1
49^†^	Porcine deltacoronavirus strain CH‐HX‐2018 spike protein gene complete cds	PDCoV/CH/HX/2018	China	3480	MK040448.1
50^†^	Porcine deltacoronavirus strain CH‐WH‐2017 spike protein gene complete cds	PDCoV/CH/WH/2017	China	3480	MK040451.1
51^†^	Pocine deltacoronavirus PDCoV/CH HN 2016 S protein (S) gene complete cds	PDCoV/CH HN 2016	China	3480	KY496312.1
52^†^	Porcine deltacoronavirus isolate CHN‐GD16‐13 spike protein gene complete cds	PDCoV/CHN‐GC16‐13	China	3480	KY078916.1
53^†^	Porcine deltacoronavirus isolate CHN‐GC16‐12 spike protein gene complete cds	PDCoV/CHN‐GC16‐12	China	3480	KY078915.1
54^†^	Porcine deltacoronavirus isolate CH/XJYN/2016 spike protein (S) gene complete cds	PDCoV/CH/XJYN/2016	China	3483	MN064712.1
55^†^	Porcine deltacoronavirus isolate GX05‐2017 spike glycoprotein gene complete cds	PDCoV/GX05‐2017	China	3480	MT505449.1
56^†^	Porcine deltacoronavirus strain CH‐DH2‐2017 spike protein gene complete cds	PDCoV/CH‐DH2‐2017	China	3480	MK040450.1
57^†^	Porcine deltacoronavirus isolate SD‐12‐2018 spike glycoprotein gene complete cds	PDCoV/SD‐12‐2018	China	3480	MN173812.1
58^†^	Porcine deltacoronavirus isolate SD‐10‐2018 spike glycoprotein gene complete cds	PDCoV/SD‐10‐2018	China	3480	MN173810.1
59^†^	Porcine deltacoronavirus isolate GX03‐2017 spike glycoprotein gene complete cds	PDCoV/GX03‐2017	China	3474	MT505447.1
60^†^	Porcine deltacoronavirus isolate GX05‐2019 spike glycoprotein gene complete cds	PDCoV/GX05‐2019	China	3483	MT505460.1
61^†^	Porcine deltacoronavirus isolate GX03‐2018 spike glycoprotein gene complete cds	PDCoV/GX03‐2018	China	3483	MT505452.1
62^†^	Porcine deltacoronavirus isolate GX03‐2019 spike glycoprotein gene complete cds	PDCoV/GX03‐2019	China	3483	MT505458.1
63^†^	Porcine deltacoronavirus isolate GX05‐2018 spike glycoprotein gene complete cds	PDCoV/GX05‐2018	China	3483	MT505454.1
64^†^	Porcine deltacoronavirus isolate GX04‐2019 spike glycoprotein gene complete cds	PDCoV/GX04‐2019	China	3483	MT505459.1
65^†^	Porcine deltacoronavirus CH‐HA2‐2017 spike protein gene complete cds	PDCoV/CH‐HA2‐2017	China	3480	MK040454.1
66^†^	Porcine deltacoronavirus strain CH‐HA3‐2017 spike protein gene complete cds	PDCoV/CH‐HA3‐2017	China	3483	MK040455.1
67^†^	Porcine deltacoronavirus isolate YUC/UICMPR/2015 spike glycoprotein (S) gene, complete cds	PDCoV/Mexico/YUC/UICMPR/2015	Mexico	3483	MK478381.1
68^†^	Porcine deltacoronavirus isolate MEXICO/OAX/UI1253CMPR/2017 spike glycoprotein (S) gene, complete cds	PDCoV/Mexico/OAX/UI1253CMPR/2017	Mexico	3483	MK478383.1
69^†^	Porcine deltacoronavirus isolate EDOMEX/UI202CMPR/2017 spike glycoprotein (S) gene, complete cds	PDCoV/Mexico/UI202CMPR/2017	Mexico	3482	MK478382.1
70^†^	Porcine deltacoronavirus isolate QRO/UI689CMPR/2017 spike glycoprotein (S) gene, complete cds	PDCoV/Mexico/QRO/UI689CMPR/2017	Mexico	3482	MK478380.1
71^†^	Porcine deltacoronavirus isolate KNU16‐08 spike protein (S) gene complete cds	PDCoV/SouthKorea/KNU16‐08	South Korea	3480	KY926511.1
72^†^	Porcine deltacoronavirus isolate SL5 spike protein gene complete cds	PDCoV/SouthKorea/SL5	South Korea	3483	KR060083.1
73^†^	Porcine deltacoronavirus isolate SL2 spike protein gene complete cds	PDCoV/SouthKorea/SL2	South Korea	3483	KR060082.1

^†^ Nucleotide sequences included for phylogenetic analysis of S gene.

### 2.5 Phylogenetic analysis

The PDCoV whole genome, ORF 1a/b and S nucleotide sequences in this study were aligned using Clustal W from MEGA X software (Kumar et. Et al., 2018). We used PDCoV genome sequences obtained from GenBank isolated in the USA (16), China (13), Japan (5), Haiti (3), South Korea (2), Vietnam (2), Thailand (2) and Laos (1). For the spike analysis, we included sequences from the USA (13), China (28), Japan (5), Mexico (*n* = 4), South Korea (3), Haiti (3), Vietnam (2), Thailand (2) and Laos (1). A general time reversible nucleotide substitution model with gamma distribution among site rate variation was used, with a maximum likelihood estimation model for phylogenetic reconstruction. Bootstrap analysis was carried out on 1000 data sets. The percentage of nucleotide sequence identity was also calculated.

## 3 RESULTS

### 3.1 Molecular detection confirms the presence of PDCoV genome in intestinal samples of pigs suffering from an enteric disease outbreak in Peru

We took advantage of a widely used qRT‐PCR assay that allows accurate differentiation of most common enteric coronaviruses in swine such as PEDV, TGEV and PDCoV. qRT‐PCR results confirmed the presence of PDCoV RNA in all (*n* = 3) samples analyzed, which tested negative to PEDV and TGEV. In addition to its qualitative feature, this assay allows relative quantification. Thus, the viral load was quantified through the amount of genetic target of PDCoV amplified during the process. Samples had low Ct values to PDCoV (Ct = 9–14) which indicated a high PDCoV viral load. From these results, we inferred that PDCoV was involved in the enteric disease with high viral titres.

### 3.2 PDCoV replicated in PK‐15 while Vero cells did not allow PDCoV propagation

Following PDCoV detection, our objective was to isolate in vitro using PK‐15 and Vero cell lines. Despite the high viral load detected by PCR, we did not see evidence of cytopathic effect during the first 5 days following inoculation in any of the cell line tested. Thus, we performed a second blind passage to evidence viral replication. Interestingly, 48 h post inoculation, cytopathic effect was observed in PK‐15 cell line, but morphological changes in Vero cell line were absent. These findings were also confirmed by qRT‐PCR. Within the major cellular changes observed are: pyknosis, cell rounding, monolayer disruption, cell detachment, which correspond to typical coronavirus cytopathic effect previously described (see Figure 1(a)). Furthermore, the immunofluorescence test in PK‐15‐infected cells confirmed the presence of PDCoV (see Figure 1(b)). Following isolation, a sample was selected and prepared for genome sequencing.

### 3.3 Whole genome and ORF 1 a/b sequencing analysis indicates that Peruvian PDCoV isolate originated from a PDCoV strain from the USA

**FIGURE 2:**
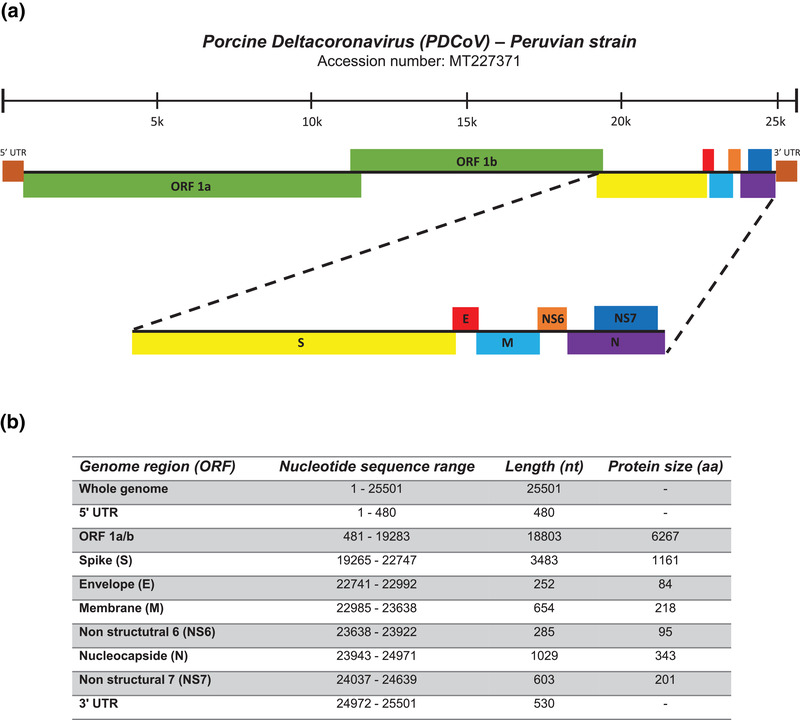
Genomic organization of the Peruvian PDCoV isolate. Whole genome sequence of the Peruvian PDCoV isolate was performed by next‐generation sequencing (NGS). PDCoV whole genome sequence is 25501 nt. Starting from 5′ end, the genome is structured as follows: 5′‐UTR, ORF 1a/b, S, E, M, NS6, N, NS7 and 3′‐UTR (a). Genomic regions, nucleotide sequence range and nucleotide and protein lengths are depicted at the bottom (b)

The nucleotide sequence of Peruvian PDCoV isolate, identified as PDCoV/Peru/isolate/2019, was submitted to GenBank under the accession number MT227371. Our Peruvian PDCoV genome follows similar patterns with other PDCoV genome sequences deposited in GenBank. Thus, this isolate is 25,501 nt in length and consists of, excluding the polyA tail: 5′‐UTR (1–480 nt), ORF1a/b (481–11,368 nt, 11,368–19,283 nt), S (19,265–22,747 nt), E (22,741–22,992 nt), M (22,985–23,638 nt), NS6 (23,638–23,922 nt), N (23,943–24,971 nt), NS7 (24,037–24,639 nt) and 3′‐UTR (24,972–25,501 nt). A graphical representation of the characterized PDCoV isolate is shown in Figure 2.

**FIGURE 3:**
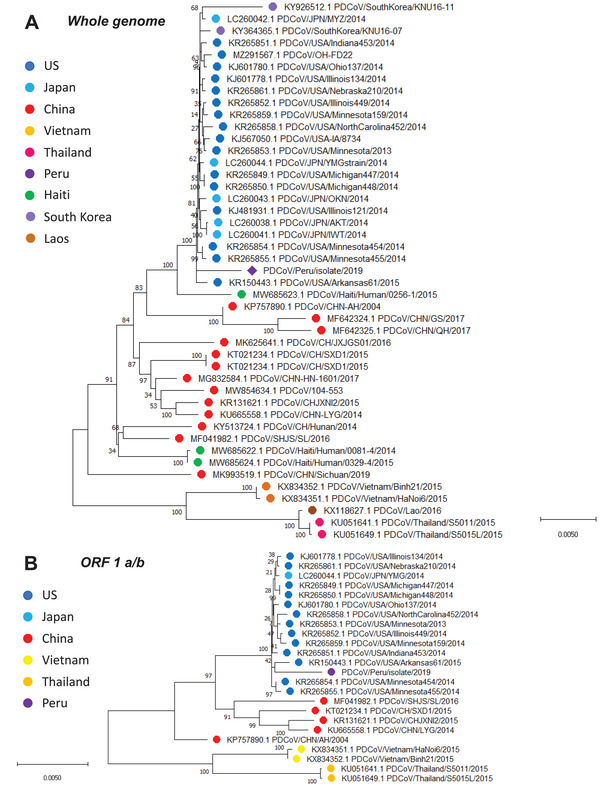
Whole genome and ORF 1a/b phylogenetic analysis of Peruvian PDCoV strain reveals its evolutionary origin from a North American PDCoV strain. For both whole genome and ORF 1a/b, the evolutionary history was inferred by using the maximum likelihood method and general time reversible model. The percentage of trees in which the associated taxa clustered together is shown next to the branches. A discrete gamma distribution was used to model evolutionary rate differences among sites. The tree is drawn to scale, with branch lengths measured in the number of substitutions per site. The whole genome analysis involved 45 nucleotide sequences, whereas the ORF 1 a/b involved 24 nucleotide sequences. Evolutionary analyses were conducted in MEGA X

Phylogenetic analysis has typically been performed using key major genes of any organism of interest. However, this analysis tends to limit the analysis to a certain gene or group genes. Conversely, whole genome sequencing offers a more complete and deeper genetic characterization compared with partial approaches. In our study, we took advantage of next generation sequencing of our PDCoV isolate to track its evolutionary origin. Our results indicated that our Peruvian strain belongs to the North American phylogroup and is closely related to a PDCoV strain from the USA isolated in 2015 (99.5% of nucleotide identity). Genetic distance of the Peruvian PDCoV strain with other PDCoV analyzed reveals high similarity between 97.1 and 99.5%. Compared with the strains from the USA, the Peruvian PDCoV has a nucleotide identity between 99.45 and 99.51%. Percentages range from 98.6 to 98.74% when compared with the Chinese strains. Finally, nucleotide identity is 97% and 97.5% for Thai and Vietnamese strains, respectively. A summary of nucleotide identity is shown in Table 2. Further analysis based on ORF 1 a/b showed identical topology to the whole genome sequence phylogenetic tree. Altogether, these results indicate that the virus detected in Peru has emerged from a North American ancestor (see Figures 3(a) and (b)). Similarly, PDCoV protein sequence analysis resembled the topology of the nucleotide analysis.

**TABLE 2:** Genetic distances of PDCoV nucleotide sequences (percentages) with the Peruvian PDCoV strain

	Whole genome	ORF 1a/b	S	E	M	NS6	N	NS7
USA	99.45–99.51	99.56–99.63	99.15–99.30	99.6	99.38–99.85	99.29–99.65	99.21–99.61	98.99–99.67
China	98.60–98.74	98.72–98.86	97.65–98.38	98.36–98.78	98.26–99.07	98.93–99.29	98.10–98.91	98.12–98.99
Japan	99.5	99.61	99.24	99.6	99.85	99.29	99.51	99.67
Thailand	97.10–97.11	97.27–97.28	95.51–95.54	99.19	98.26	98.57	96.83	97.04
Vietnam	97.46–97.5	97.50–97.53	95.95–96.05	99.6	98.91	98.93	98.61	98.12
Mexico	–	–	98.65–99.21	–	–	–	–	–

### 3.4 Phylogenetic analysis of Peruvian PDCoV S gene shows close relationship to the Mexican PDCoV within the North American phylogroup

**FIGURE 4:**
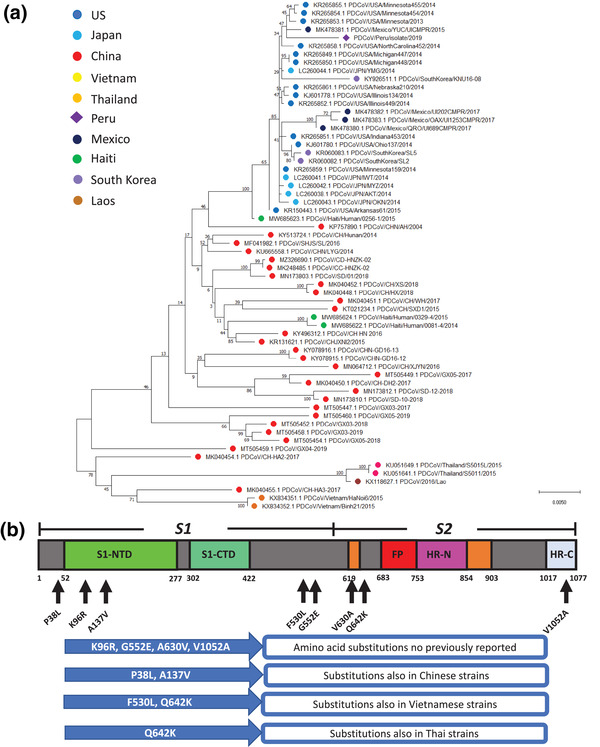
Phylogenetic analysis of S gene reveals that Peruvian PDCoV strain has a close relationship with a Mexican strain within the North American phylogroup. The evolutionary history was inferred by using the maximum likelihood method and general time reversible model. The percentage of trees in which the associated taxa clustered together is shown next to the branches. A discrete gamma distribution was used to model evolutionary rate differences among. The tree is drawn to scale, with branch lengths measured in the number of substitutions per site. This analysis involved 62 nucleotide sequences. Evolutionary analyses were conducted in MEGA X (a). Schematic representation of the porcine deltacoronavirus S protein highlighting the amino acid substitutions found in the present study. Black arrows represent the site of these substitutions. A multiple amino acid sequence alignment of S protein was performed using Clustal W in MEGA X. Eight amino acid modifications were detected within the Peruvian sequence compared to others (b)

S gene is one of the most variable genes among coronaviruses. This is due to its function in cell attachment and viral replication. This high polymorphism makes the S gene a powerful tool to estimate the evolutionary relationship among virus strains belonging to the same genetic group. Hence, we performed a phylogenetic analysis using the PDCoV S nucleotide sequences publicly available with our Peruvian S nucleotide sequence to evaluate their evolutionary distance (see Figure 4(a)). Our results indicate that our PDCoV isolate has close relationship to the Mexican PDCoV strains (98.65–99.21% of nucleotide identity) within the North American phylogroup. Similarly, S protein phylogenetic analysis reveal similar evolutionary relationship among strains analyzed. Yet, S phylogenetic analysis evidence that Peruvian PDCoV has a certain degree of divergency from the North American phylogroup.

### 3.5 Peruvian PDCoV Spike amino acid sequence reveals unique substitutions compared to other PDCoV strains

As we observed multiple changes at the nucleotide level in the S gene, we were interested in evaluating whether these changes represent modifications at the protein level. Thus, we found multiple changes in the S protein sequence (see Figure 4(b)). Most relevant changes are K96R, G552E, A630V and V1052A that represent unique variations compared with other PDCoV strains. Other amino acid changes have been also found in Chinese strains such as P38L and A137V. We also detected F530L, like the one found in the Vietnamese strains. Furthermore, the Peruvian strains have a Q642K, like those in Vietnamese and Thai strains. These results provide evidence that PDCoV has undergone to unique changes that indicate a degree of genetic diversity in the Peruvian PDCoV strain. Based on previous studies of PDCoV Spike protein characterization (Shang et al., 2018), amino acid substitutions observed here are located randomly across both S protein subunits (S1 and S2) but none of them is located in the RBD region (S1‐CTD).

## 4 DISCUSSION

PDCoV is one of the most recent and relevant coronaviruses of swine industry. It represents a major threat for swine productivity, and it is responsible for large economic losses worldwide. Yet, PDCoV remains poorly studied despite major efforts made recently. In Peru, multiple cases of enteric disease have occurred; however, these cases are not properly addressed and frequently misdiagnosed. Hence, this study represents the first report of isolation and phylogenetic characterization of a Peruvian PDCoV isolate using the whole genome sequence and its major S protein, revealing unique aspects compared to other PDCoV strains.

Although multiple studies have proven that ST, LLC‐PK (Hu et al., 2015) and IPI‐2I (Zhang et al., 2019) are the primary option for PDCoV isolation, we showed that PK‐15 might be a valid alternative when is needed. This is also demonstrated by Jiang et al. (2019), in the assessment of innate immune activation following PDCoV infection. Nevertheless, it is possible that efficacy in viral replication might vary among different cell lines and PK‐15 might not be as permissive as others. This should be considered when viral infectivity is the main objective. In addition, since we detected viral replication in the presence of trypsin, we speculate that PK‐15 cells might offer a compared PDCoV permissibility in LLC‐PK while differing from that observed in ST. Furthermore, our viral isolation findings contrasted with those detected by qRT‐PCR. This provided evidence that a large proportion of viral particles were unable to replicate into a cell line support. This has also been reported by others indicating that low viral isolation rates might be attributed to sample degradation and viral viability (Hu et al., 2015). On the other hand, multiple authors have shown that successful viral isolation are due to other factors such as cell line permissibility and enzyme treatment (Jung et al., 2016; Yang et al., 2020; Zhao et al., 2019). In our study, it remains unclear whether trypsin concentration (10 μg/ml) might have played a role in the lack of viral replication in Vero cells. Additional studies, at different trypsin concentration, will elucidate Vero cell line permissibility to PDCoV. Interestingly, we did not observe cell toxicity in our assays, as that was absent following the first 5 days of inoculation and PDCoV cytopathic effect was evidenced in the second passage. These results contrast with other claims that cell toxicity is common during PDCoV isolation using intestinal content or foecal samples (Hu et al., 2015). Nevertheless, further studies are required to clarify the implications of cell permeability to PDCoV in viral replication and its effects on clinical presentation.

Whole genome analysis revealed that our isolate was closely related to North American strains. The close relationship within the Peruvian and the North American strains indicate they share a common phylogenetic ancestor and revealed that the Peruvian isolate emerged from a strain from the USA. In 2019, Perez‐Rivera et al. reported the first phylogenetic analysis of PDCoV in Mexico, focusing the analysis on the S gene nucleotide sequence, and no report of PDCoV whole genome sequence was made (Pérez‐Rivera et al., 2019). Thus, we were unable to evaluate the phylogenetic relationship using the whole genome sequence of PDCoV from Peru and Mexico. We believe that this would add deeper understanding about its appearance in Peru and contribute to its epidemiology in South America.

Due to its high variability, S gene nucleotide sequences have been used to estimate the genetic relationship of PDCoV strains worldwide. Perez‐Rivera et al. (2019) demonstrated that Mexican PDCoV formed two clades: the group including strains isolated in 2015 and those PDCoV isolated in 2017. In our study, phylogenetic analysis using S gene nucleotide sequences reveals that Peruvian PDCoV grouped closely to the Mexican strain isolated in 2015 within the North American phylogroup. This indicates a close relationship among PDCoV strains from Mexico, the USA and Peru, sharing a common ancestor and evidencing a dissemination route of PDCOV from North America to South America. Interestingly, multiple non‐silent mutations were found in the Peruvian PDCoV strain compared with other genomes. Even though some of these mutations have been described in other PDCoV strains, some are unique to the Peruvian isolate, revealing that this isolate has undergone phenotypical changes after its emergence in North America. Although these substitutions were neither located in critical regions of glycosylation sites nor in the RBD region (S1‐CTD), they might have influence in ligand/receptor interaction. Further studies are required to clarify whether these modifications have implications in the pathobiology and development of the clinical disease.

To date, it is unclear how PDCoV was introduced in Peru. However, there is a long history of commerce between Peru and North American countries that has expanded in recent years. The National Service of Animal Health in Peru (SENASA) reported the import of a large number of purebred animals (∼150 tons) during the 2014 and 2018 period. Furthermore, Peru imports feed ingredients for swine farms mainly from the USA (MINAGRI, 2020). Altogether, this might explain the possible routes for PDCoV entrance into the country, similar to that described for other viruses of importance for swine industry (Dee et al., 2018; Ramírez et al., 2019). Interestingly, there is no report of PDCoV in other South American countries so the introduction from those is unlikely. Nevertheless, further studies are needed to understand the epidemiology of this disease in Peru and its relationship to other countries.

In conclusion, Peruvian PDCoV strain was successfully sequenced, isolated and phylogenetically analyzed demonstrating that this isolate has been derived from a strain identified in the USA. To our knowledge, this is the first report of a PDCoV strain detected in South America and offers new insights about the epidemiology of PDCoV worldwide.

## CONFLICT OF INTEREST

All authors have declared no conflict of interest.

## ETHICAL STATEMENT

The authors confirm that the ethical policies of the journal, as noted on the journal's author guidelines page, have been adhered to. No ethical approval was required as research samples were obtained in accordance with guidelines from the Peruvian National authorities in animal health.

## Data Availability

The data that support the findings of this study were submitted to the GenBank database (https://www.ncbi.nlm.nih.gov/genbank/) with accession number MT227371 for the Peruvian strain of PDCoV obtained.
